# Native T_1_ mapping identifies subclinical left ventricular myocardial t1 abnormality in patients with atrial fibrillation referred for pulmonary vein isolation

**DOI:** 10.1186/1532-429X-17-S1-P351

**Published:** 2015-02-03

**Authors:** Shingo Kato, Sébastien Roujol, Jihye Jang, Tamer A Basha, Sophie Berg, Kraig V Kissinger, Goddu Beth, Warren J Manning, Reza Nezafat

**Affiliations:** 1 Beth Israel Deaconess Medical Center, Boston, MA, USA; 2 Yokohama City University Hospital, Yokohama, Japan

## Background

Native T_1_ mapping has emerged as a noninvasive magnetic resonance imaging (MRI) method to assess the diffuse myocardial fibrosis without an exogenous contrast agents. To date, no data are available regarding native left ventricular (LV) T_1_ relaxation time in AF patients referred for pulmonary vein isolation (PVI). The purpose of this study was to compare native myocardial T_1_ value between AF patients undergoing PVI and control subjects.

## Methods

Thirty one AF patients referred for PVI (58±8 yrs; 23 m) and 17 control subjects (50±15 yrs; 13 m) were retrospectively identified. All patients were in sinus rhythm during the CMR scan. No AF patient had a history of myocardial infarction but 7 of 31 (23%) AF patients had history of hypertension. Non-contrast T_1_ mapping images were acquired using a Modified Look-Locker imaging sequence (MOLLI) in 3 short-axis planes (basal, mid and apical slices) using an ECG-triggered single-shot acquisition with a balanced SSFP readout (TR 3.1; TE 1.5; FA 35°; FOV 360×337 mm^2^; acquisition matrix 188×135; voxel size 1.9 × 2.5 mm^2^; slice thickness 8 mm). Late gadolinium enhanced (LGE) MRI was acquired to evaluate for myocardial scar.

## Results

LV ejection fraction was similar between groups (AF: 60±7%, controls: 58±7%, p=0.27). No LGE myocardial scar was observed in any AF patient or control subject. Myocardial native T_1_ was significantly elevated in AF patients (1106±48 msec vs 1059±33 msec, p=0.001). Multivariate linear regression analysis selected presence of AF as an independent and significant predictor of elevated native T_1_ after adjustment for age and gender (OR: 25.8, 95%CI: 2.9 - 228.3, p=0.003).

## Conclusions

Our study suggests that there are subclinical differences (vs. normal controls) in native myocardial T_1_ between AF patients referred for PVI. These data suggests that T_1_ mapping may be useful for early detection of subclinical LV myocardial abnormalities in the AF population.

## Funding

Shingo Kato, MD receives scholarship from Banyu Life Science Foundation International.

**Figure 1:**
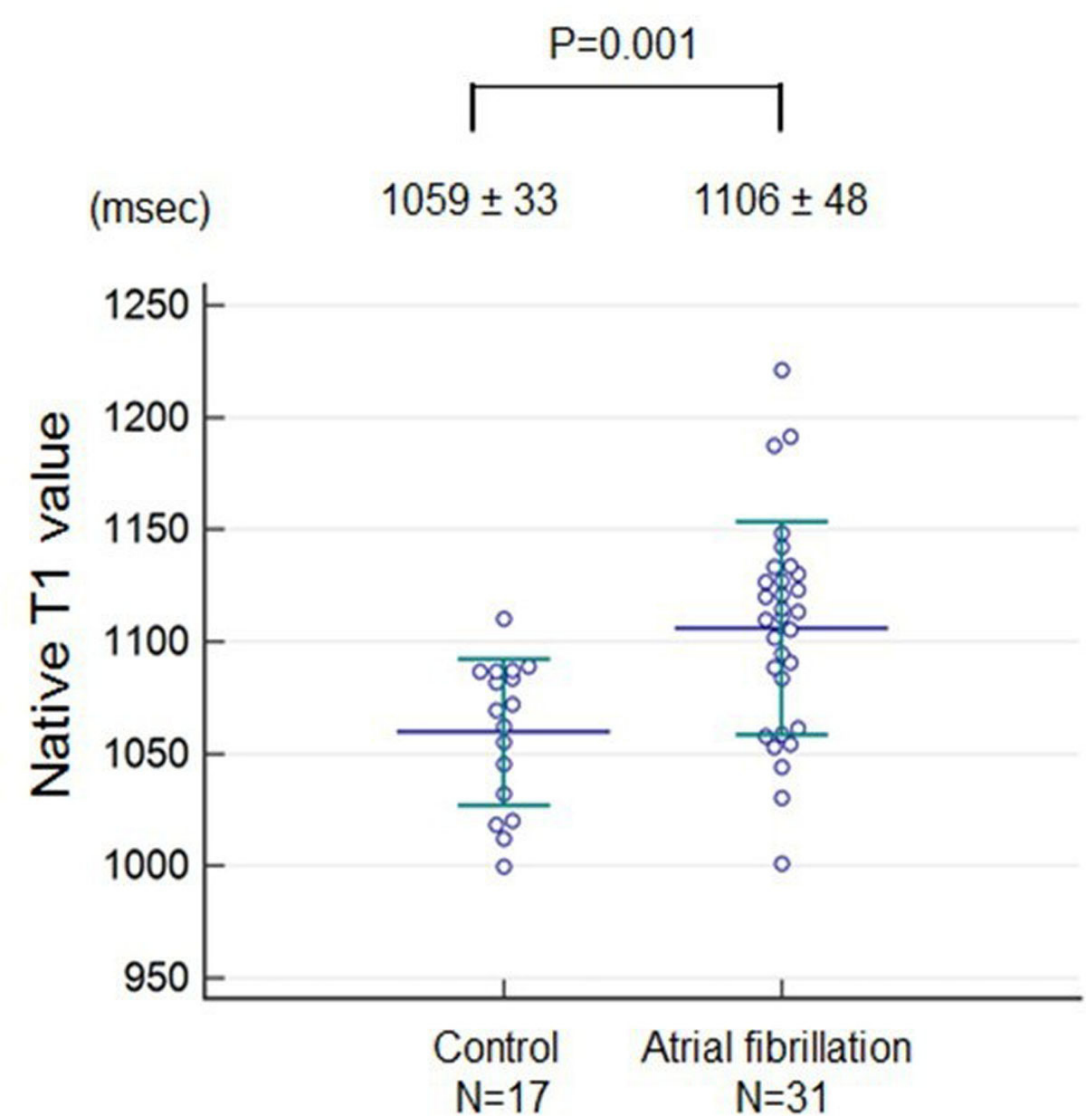
**Comparison of native T1 value between atrial fibrillation patients and controls.** Native T1 value was significantly elevated in atrial fibrillation patient compared with control subject.

